# Gut Microbiota Regulate Saturated Free Fatty Acid Metabolism in Heart Failure

**DOI:** 10.1002/smsc.202300337

**Published:** 2024-07-08

**Authors:** Gulinigaer Tuerhongjiang, Manyun Guo, Xiangrui Qiao, Junhui Liu, Wen Xi, Yuanyuan Wei, Peining Liu, Bowen Lou, Chen Wang, Lizhe Sun, Xiao Yuan, Hui Liu, Ying Xiong, Yunlong Ma, Hongbing Li, Bo Zhou, Lijuan Li, Zuyi Yuan, Yue Wu, Jianqing She

**Affiliations:** ^1^ Cardiovascular Department First Affiliated Hospital of Xi'an Jiaotong University Xi'an Shaanxi 710061 China; ^2^ Key Laboratory of Environment and Genes Related to Diseases Ministry of Education Xi'an Shaanxi 710061 China; ^3^ Diagnostic Department First Affiliated Hospital of Xi'an Jiaotong University Xi'an Shaanxi 710061 China; ^4^ Department of Cardiology Second Affiliated Hospital Zhejiang University School of Medicine Hangzhou 310058 China; ^5^ Biobank First Affiliated Hospital of Xi'an Jiaotong University Xi'an Shaanxi 710061 China; ^6^ Respiratory Department First Affiliated Hospital of Xi'an Jiaotong University Xi'an Shaanxi 710061 China; ^7^ Cardiovascular Department Wuzhong People's Hospital Ningxia 215128 China

**Keywords:** *Clostridium sporogenes*, free fatty acids, gut microbiota, heart failure

## Abstract

Aims: Heart failure (HF) is associated with profound changes in cardiac metabolism. At present, there is still a lack of relevant research to explore the key microbiome and their metabolites affecting the progression of HF. Herein, the interaction of gut microbiota and circulating free fatty acid (FFA) in HF patients and mice is investigated. Methods and Results: In HF patients, by applying metagenomics analysis and targeted FFA metabolomics, enriched abundance of *Clostridium sporogenes* (*C.sp*) in early and late stage of HF patients, which negatively correlated to saturated free fatty acid (SFA) levels, is identified. KEGG analysis further indicates microbiota gene enrichment in FFA degradation in early HF, and decreased gene expression in FFA synthesis in late HF. In HF mice (C57BL/6J) induced by isoproterenol (ISO), impaired intestinal permeability is observed, and decreased fecal *C.sp* and increased SFA are further validated. At last, by supplementing *C.sp* to ISO‐induced HF mice, the cardiac function, fibrosis, and myocardial size are partially rescued, together with decreased circulating SFA levels. Conclusions: *Clostridium* abundance is increased in HF, compensating cardiac function deterioration via downregulation of circulating SFA levels. The results demonstrate that the gut microbiota–SFA axis plays an important role in HF protection, which may provide a strategic advantage for the probiotic therapy development in HF.

## Graphical abstract

The exploration of metabolic characteristics in heart failure (HF) patients at different stages through metagenomics and targeted fatty acids metabolomics has revealed the significant role of *Clostridium sporogenes* (*C.sp*)–saturated free fatty acid–cardiac function axis in HF, which at least partially contributes to HF progression. Probiotic intervention with *C.sp* may bring a new breakthrough in the treatment of HF.

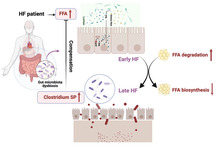

## 1 Introduction

Heart failure (HF) is a complex clinical syndrome, defined as any structural or functional impairment of ventricular filling or ejection capability.^[^ ^1^ ^]^ It has been proven that cardiac metabolism disturbances are related to cardiac function deterioration and HF progression, as heart is in critical need for a constant supply of energy.^[^ 2, 3, 4, 5 ^]^ However, previous metabolic cardiovascular research has usually relied on specific metabolic pathways in a narrowly focused fashion. Few studies have evaluated interactions between gut microbiota and circulating free fatty acid (FFA) in HF.

Fatty acids, which serve as the principal source of energy for the adult heart, are integral to the process of β‐oxidation within cardiac energy metabolism.^[^ ^6^ ^]^ It has been widely demonstrated that saturated free fatty acids (SFA) are recognized as a known risk factor for cardiovascular diseases (CVD).^[^ ^7^ ^]^ Conversely, polyunsaturated fatty acids (PUFA) have been linked to a reduction in CVD.^[^ ^8^ ^]^ The impact of monounsaturated fatty acids (MUFA) remains inconclusive and requires further investigation.^[^ ^9^ ^]^ Moreover, it is reported that replacing SFA with PUFA or MUFA can have a positive effect on CVD.^[^ 10, 11 ^]^ The relationship between FFA utilization and metabolism during HF has been investigated extensively. In HF, myocardium caused by long‐term diabetes, increased lipid droplets accumulation has been identified, along with activated endoplasmic reticulum stress response and apoptosis, suggesting loss of utilization for FFA in HF patients.^[^ 12, 13 ^]^ Moreover, essential depletion of cellular FFA has been observed in animal models of end‐stage HF with reserved ejection fraction.^[^ ^14^ ^]^ It is also reported that higher levels of circulating long‐chain SFA are associated with lower risk of incident HF in cohort study among older adults.^[^ ^15^ ^]^ Recent studies have shown diminished FFA oxidation in experimental HF models with a shift toward oxidation of ketone bodies.^[^ 16, 17, 18 ^]^ However, conflicting evidence and limited data on FFA metabolism in different stages of HF mandate further metabolic profiling and mechanism validation studies.

Advances in our understanding of the interaction between gut microbiota and circulating metabolites have expanded our insights into how microbial composition and associated metabolites affect the human host. The relationship between gut microbiota and microbiota‐derived metabolites is reciprocal.^[^ ^19^ ^]^ Fatty acids have the potential to induce changes in both the structure and function of gut microbiota.^[^ ^19^ ^]^ Short‐chain fatty acids (SCFAs) are predominantly generated through the saccharolytic fermentation of nondigestible carbohydrates by gut bacteria.^[^ ^20^ ^]^ It has been established that gut microbiota are capable of producing long‐chain fatty acids.^[^ 21, 22, 23 ^]^ However, the field of microbial diversity and metabolic disturbances in HF is still emerging.^[^ 24, 25, 26 ^]^ It is hypothesized that bowel wall edema and impairment in barrier function during HF lead to translocation of gut microbiota components into the host circulation, causing a heightened systemic inflammatory state.^[^ ^27^ ^]^ Recent study has shown that the gut microbiota signature in chronic HF is characterized by low bacterial richness and depletion of bacteria with butyrate‐producing potential, hypothetically leading to persistent T‐cell activation.^[^ ^24^ ^]^ Moreover, a number of metabolic pathways, including trimethylamine–trimethylamine *N*‐oxide pathway, SCFA pathway, and bile acid pathway have been revealed to be involved in HF.^[^ ^28^ ^]^ Of note, interaction between FFA and gut microbiota has been identified in the pathogenesis of HF by demonstrating that the gut microbiota could produce SCFAs, interacting with FFA receptor 2 and 3.^[^ 29, 30 ^]^ However, comprehensive FFA profile alteration and underlying gut microbiota dysbiosis modulation in HF remain to be elucidated. At present, there is still a lack of relevant research to explore the key microbiome and their metabolites affecting the progression of HF.

In this study, we have recruited HF patients in the early or late stage of disease onset, aiming to identify intercorrelation of gut microbiota and circulation FFA in HF patients. We hypothesize that substantial circulation FFAs excess exist in HF, due to sympathetic nerve excess activation, systemic inflammation, and glucose intolerance. Differential gut microbiota among HF patients in different stages might, in turn, orchestrate serum FFA profile and cardiac function. The in vivo verification studies further demonstrate that the *Clostridium sporogenes* (*C.sp*)–FFA axis plays an important role in host cardiac function mitigation, which may provide a strategic advantage for the next generation of HF drug and probiotics development.

## 2 Results

### 2.1 Gut Microbiota Analysis Identified Differential Microbiome in Early and Late HF Patients

**Figure 1:**
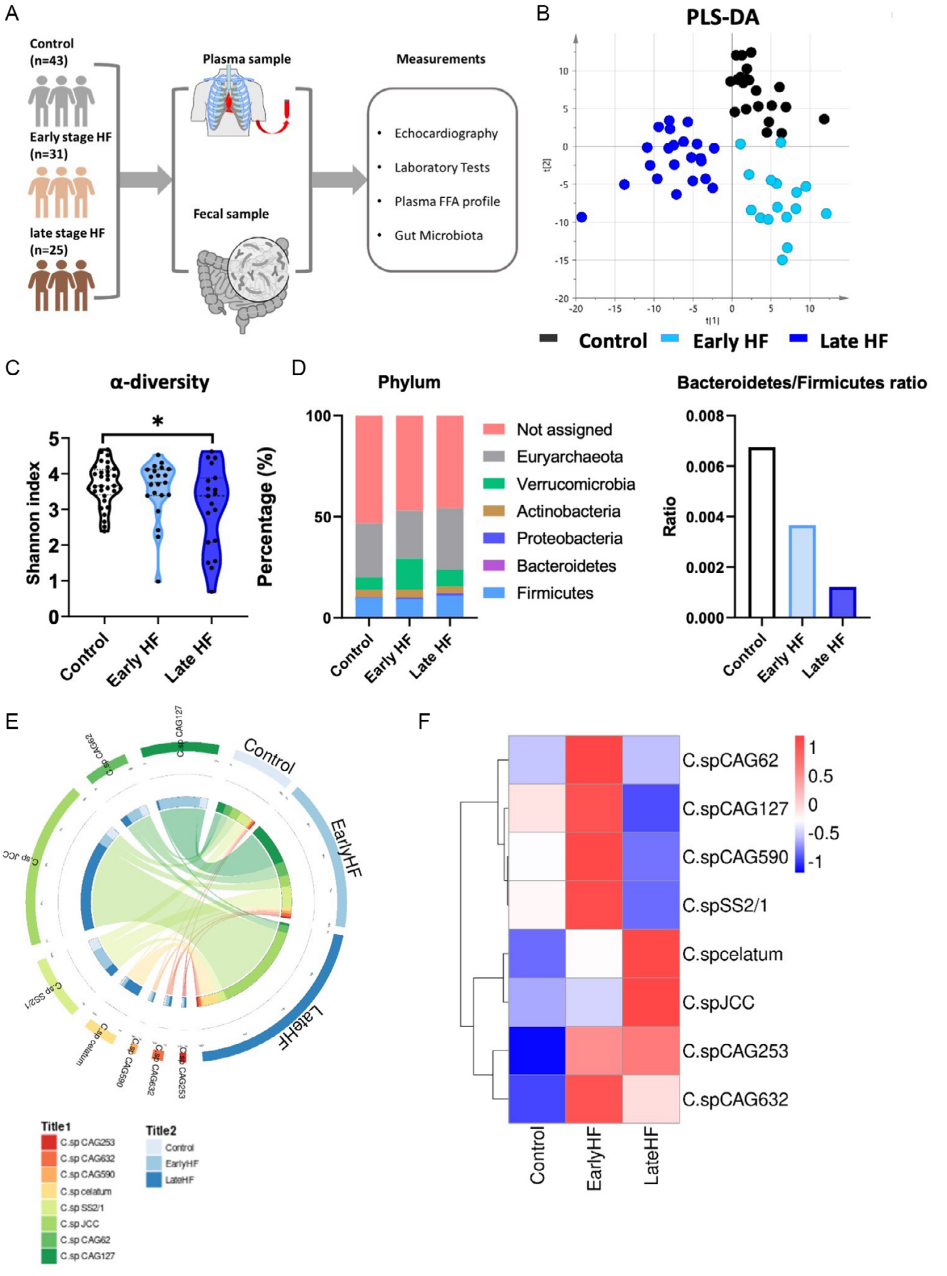
Gut microbiota analysis identified differential microbiome in early and late HF patients. A) Flowchart of the study. B) Projection plots of gut microbiota from PLS‐DA for the control (black dots), early HF (light blue dots), and late HF (dark blue dots) patients. C) *α* diversity of gut microbiota in the control, early, and late HF patients. The *α* diversity in late HF patients was significantly reduced as compared to control and early HF patients (*, *P* < 0.05). D) Stack plots of the gut microbiota at phylum level and the ratio of *Firmicutes* and *Bacteroidetes* in control, early HF, and late HF patients. E) Circus plot showing the distribution of the significantly altered *Clostridium* species in control, early HF, and late HF patients. F) Heatmap plot showing the levels of the significantly altered *Clostridium* species in control, early HF, and late HF patients.

We included a cohort of 99 adults. The schematic flowchart, which illustrated how participants were enrolled and samples were collected, is shown in **Figure** **1**A. Clinical features were generally comparable in age, sex, heart rate, blood pressure, liver function, and thyroid function among control, early HF, and late HF patients. Significant differences were noted in left ventricular ejection fraction (LVEF), N‐terminal brain natriuretic peptide (NT‐proBNP), creatinine, and high‐density lipoprotein cholesterol (HDL‐C) (**Table** **1**). To reduce potential bias to gut microbiota and metabolomics analysis, equivalent percentage of hypertension and diabetes patients was also included in the control group.

**Table 1:** Baseline characteristics.

	Control (*n* = 43)	Early HF (*n* = 31)	Late HF (*n* = 25)	*P*
Age [years]	57.07 ± 11.96	60.73 ± 9.14	66.00 ± 9.21	ns
Female [%]	29.79%	29.79%	23.08%	
Hypertension [%]	44.68%	55.56%	46.15%	
Diabetes [%]	21.28%	25.93%	30.77%	
HR [bpm]	72.86 ± 12.16	70.19 ± 11.72	77.50 ± 14.94	ns
sBP [mmHg]	126.45 ± 15.36	130.67 ± 19.43	124.25 ± 17.55	ns
dBP [mmHg]	74.71 ± 11.13	71.00 ± 9.67	75.50 ± 13.03	ns
EF [%]	67.79 ± 5.10	66.81 ± 6.02	35.38 ± 5.34	<0.001
AST [U L^−1^]	20.99 ± 6.11	21.16 ± 6.98	21.81 ± 6.11	ns
ALT [U L^−1^]	22.04 ± 13.89	24.64 ± 15.27	29.88 ± 18.25	ns
CRE [mg dL^−1^]	61.64 ± 15.24	63.44 ± 11.59	82.27 ± 20.94	<0.05
UA [mmol L^−1^]	295.27 ± 91.37	299.00 ± 75.84	359.82 ± 82.60	ns
CHOL [mg dL^−1^]	4.03 ± 1.15	3.72 ± 0.85	4.20 ± 1.24	ns
TG [mg dL^−1^]	1.66 ± 1.05	1.82 ± 1.13	1.50 ± 0.55	ns
HDLC [mg dL^−1^]	1.11 ± 0.32	0.93 ± 0.20	0.89 ± 0.23	<0.001
LDLC [mmol L^−1^]	2.31 ± 0.89	2.17 ± 0.71	2.69 ± 1.19	ns
NT‐proBNP [ng mL^−1^]	52.06 ± 33.15	602.35 ± 531.93	4451.29 ± 8709.02	<0.001
HbA1C [%]	5.83 ± 1.12	6.06 ± 0.83	6.10 ± 0.74	ns
T3 [ng mL^−1^]	1.23 ± 0.21	1.16 ± 0.24	1.13 ± 0.27	ns
T4 [μg dL^−1^]	7.75 ± 1.89	6.84 ± 1.76	7.49 ± 2.27	ns
TSH [μIU mL^−1^]	2.71 ± 2.01	3.12 ± 3.57	3.48 ± 3.55	ns

To investigate the gut microbiota alteration among control, early, and late HF patients, we utilized metagenomics and deep sequencing for better classification and in‐depth detection of altered microbiota in genus and species levels among three groups. Figure 1B shows that the partial least square discriminant analysis (PLS‐DA) was able to distinguish control, early, and late HF patients. The *α* diversity of gut microbiota in late HF was significantly reduced, in accordance with previous study;^[^ ^31^ ^]^ while no difference was observed in early HF group compared with control group (Figure 1C). Of note, although the phylum percentage was generally comparable among three groups, the ratio of *Firmicutes* and *Bacteroidetes* decreased from early HF to late HF (Figure 1D). We found that the *Clostridium* levels were considerably enriched at the species levels (Figure 1E,F; Figure S1B,C,D, Supporting Information). Thus, our results indicated that the composition of gut microbiota changed significantly and that *Clostridium*, especially *C.sp*, were substantially enriched starting from early HF and lasting to late HF.

### 2.2 KEGG Module of Gut Microbiota Identified Enhanced FFA Degradation in Early HF and Reduced FFA Biosynthesis in Late HF Patients

**Figure 2:**
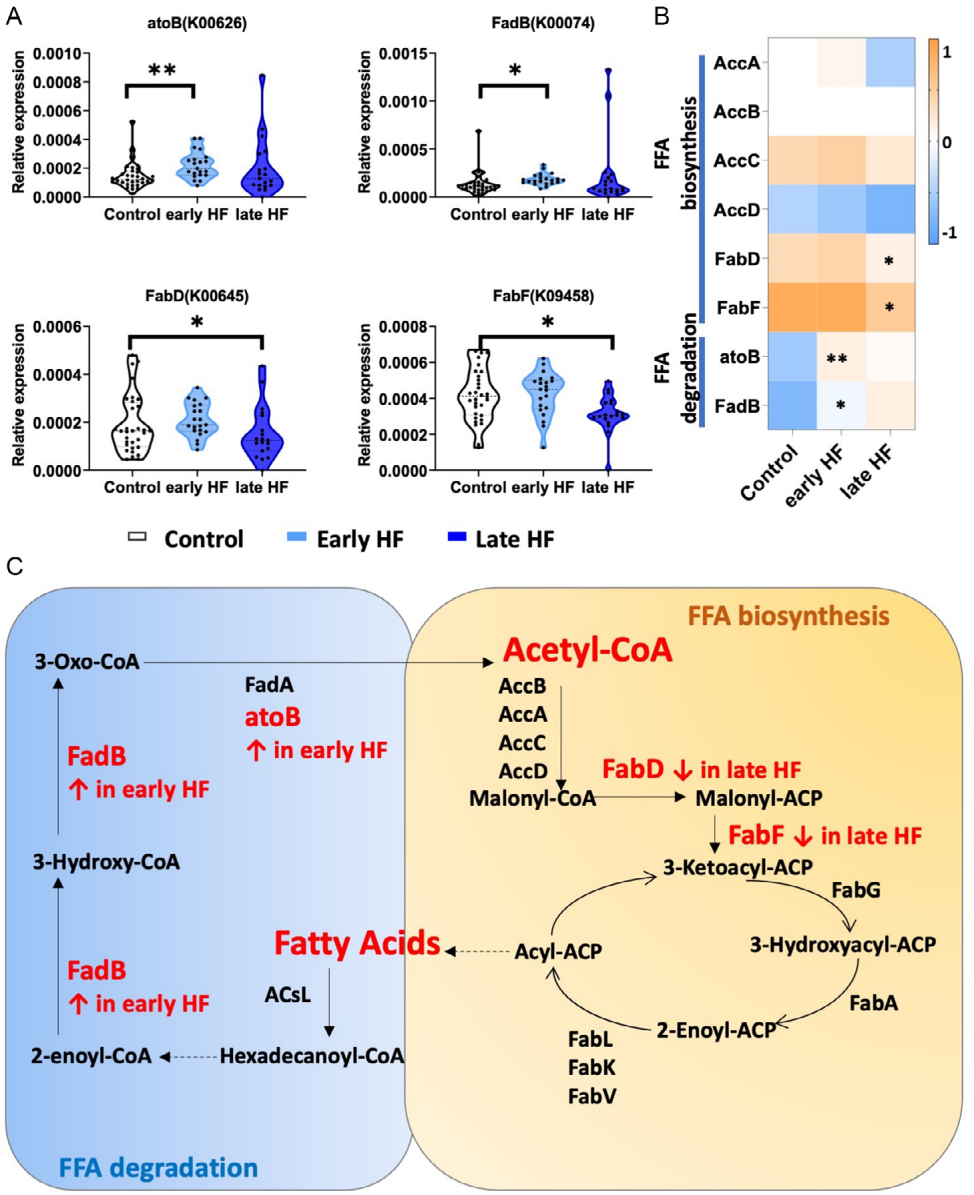
KEGG module of gut microbiota identified enhanced FFA degradation in early HF and reduced FFA biosynthesis in late HF patients. A) Relative expression of atoB, FadB, FabD, and FabF in control, early, and late HF patients. B) Heatmap showing the relative levels of the genes involved in FFA biosynthesis and FFA degradation (*, *P* < 0.05; **, *P* < 0.01). C) FFA biosynthesis and degradation pathway with respective metabolites and key enzymes.

As the metagenomic analysis revealed potential gut microbiota alteration in early and late HF patients, we conducted further investigation into the functional alterations associated with gut microbiota dysregulation by utilizing the KEGG module. Stack plots of the KEGG metabolism indicated that the general metabolism pathways including amino acids, FFA, bile acids, and so on were comparable among the three groups (Figure 1SA, Supporting Information). We then explored the significantly differentially expressed genes in three groups. It was identified that the acetyl‐CoA C‐acetyltransferase (atoB, K00626) and 3‐hydroxybutyryl‐CoA dehydrogenase (FadB, K01825) involved in fatty acid degradation were increased more markedly in early HF, whereas the *S‐*malonyl transferase (FabD, K00645) and 3‐oxoacyl‐[acyl‐carrier‐protein] synthase II (FabF, K09458) were significantly reduced in late HF (**Figure** **2**A). Heatmap for the relative gene expressions involved both in FFA biosynthesis and degradation is presented in Figure 2B, indicating that a gradual decrease of FFA biosynthesis and increase of FFA degradation occurred during the disease progression from early HF to late HF (Figure 2C). Taken together, KEGG analysis indicated that altered gut microbiota genes were inclined to correlate to FFA degradation in early patients and FFA synthesis in late HF patients.

### 2.3 Circulating FFA Levels Were Significantly Altered and Negatively Correlated to Myocardial Function in HF Patients

**Figure 3:**
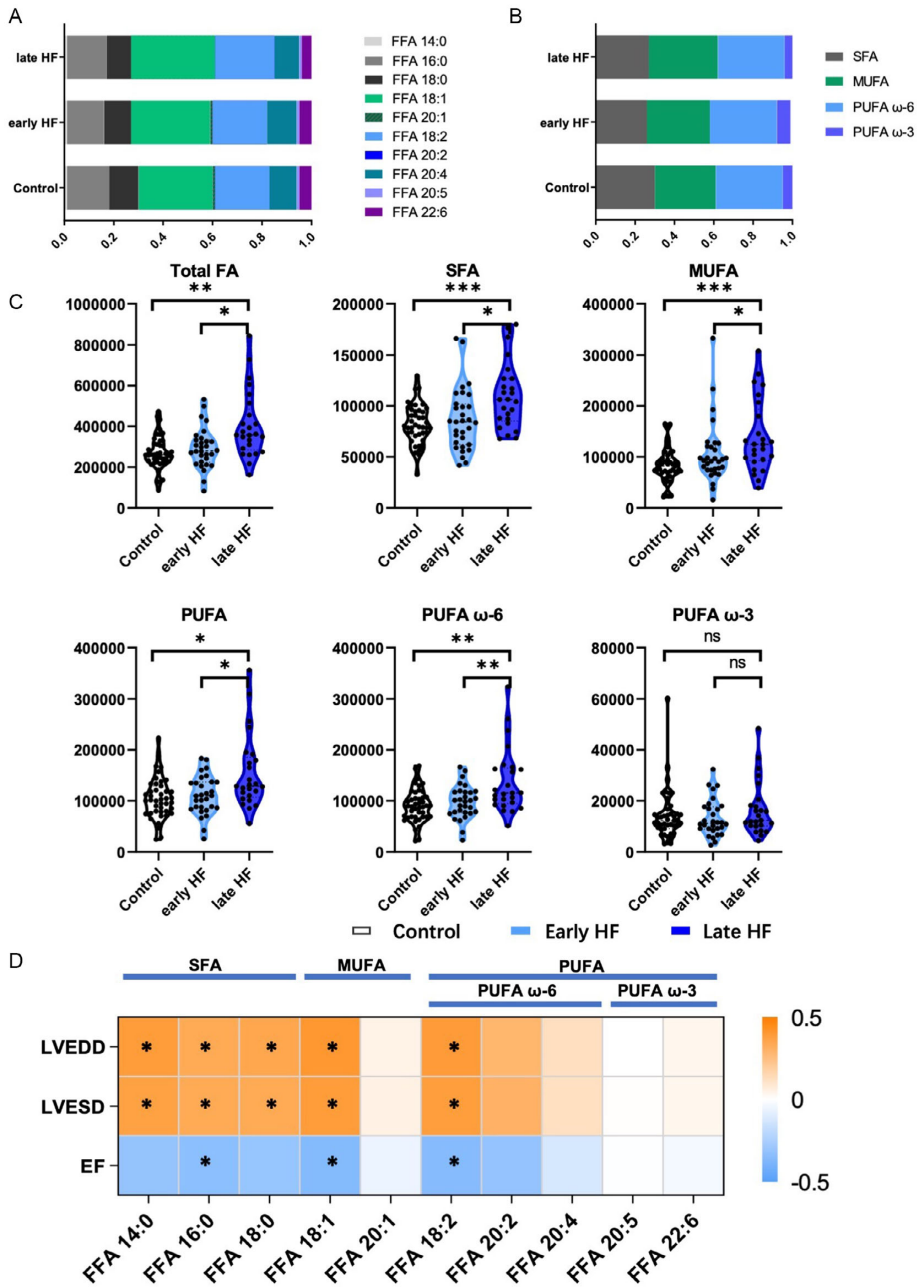
Circulating FFA levels were significantly altered and negatively correlated to myocardial function in HF patients. A) Percentage of the individual FFA levels in the control, early, and late HF groups. B) Percentage of the serum SFA, MUFA, and PUFA‐*ω*3 and PUFA‐*ω*6 profiles in the control, early, and late HF groups. C) Levels of the serum total FA, SFA, MUFA, and PUFA in the control, early, and late HF groups. D) Heatmap showing correlation between serum SFA, MUFA, and PUFA profiles and LVESD, LVEDD, and LVEF (ns, not significant; *, *P* < 0.05; **, *P* < 0.01; ***, *P* < 0.001).

As the metagenomics and KEGG enrichment analysis targeted the dysregulated FFA metabolism among HF patients, we carried out an exploration of the circulating FFA levels in patients enrolled. Toward this end, we applied targeted metabolomics to test serum SFA, MUFA, and PUFA profiles in the enrolled participants. A total of three kinds of SFAs including FFA14:0, FFA16:0, and FFA18:0, two MUFAs including FFA18:1 and FFA 20:1, three *ω*6 PUFAs including FFA18:2, FFA20:2, and FFA 20:4, and two *ω*6 PUFAs including FFA20:5 and FFA20:6 were evaluated (Figure S2, Supporting Information). The composition percentages of individual FFAs (**Figure** **3**A) and FFA classes (Figure 3B) were generally unaltered in three groups. whereas serum total FA, SFA, and MUFA were significantly increased in late HF, but not in early HF (Figure 3C). Serum *ω*6 PUFAs were also increased in late HF, but the *ω*3 PUFA levels did not show any obvious difference (Figure 3C). To further investigated the correlation between FFA levels and cardiac function, we applied echocardiography for each patient enrolled; left ventricular end diastolic and systolic diameter (LVESD and LVEDD) as well as LVEF were measured and recorded. As shown in Figure 3D, the FFA 14:0, FFA 16:0, and FFA 18:0 in SFAs were significantly positively correlated to LVESD and LVEDD, while significantly negatively correlated to LVEF. Same correlations were observed in FFA 18:1 in MUFA and FFA 18:2 in PUFA. Also, the whole SFA and MUFA were identified to be positively correlated to LVESD and LVEDD, and negatively correlated to LVEF (Figure S3, Supporting Information). In general, the higher SFA and MUFA, the worse the cardiac function in HF patients.

### 2.4 Clostridium Negatively Correlated with Circulating SFA Levels

**Figure 4:**
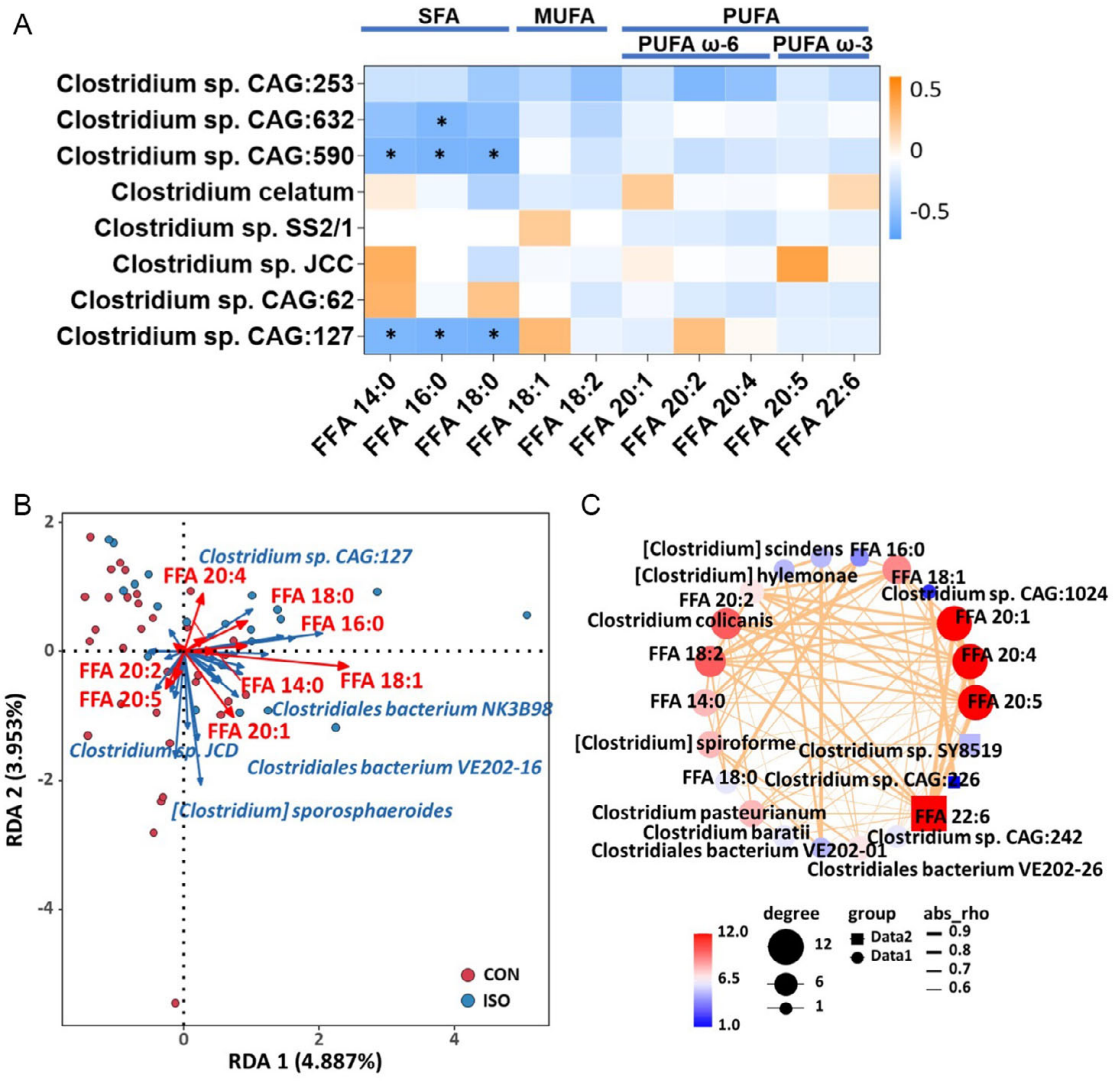
*Clostridium* negatively correlated to circulating SFA levels. A) Correlation between significantly increased *Clostridium* species and serum FFA profile (*, *P* < 0.05). B) RDA showing the relationship between *Clostridium* and FFAs. C) Correlation network between *Clostridium* and FFAs.

As it was established that the gut microbiota in HF patients potentially regulated host FFA metabolism, we then asked the question whether specific gut microbiota species were enrolled in FFA regulation during the disease progression of HF. To this end, we investigated the correlation between gut microbiota species and serum FFA profile (**Figure** **4**A). Interestingly, the *Clostridium* species negatively correlated with the SFA levels, including FFA 14:0, FFA 16:0, and FFA 18:0, with *C.sp* CAG:632, *C.sp* CAG:590, and *C.sp* CAG:127 showed significance. Redundancy analysis (RDA) and correlation network further exhibited the relationship between *Clostridium* and FFAs (Figure 4B,C). Taken together, we speculated that *C.sp* species increased in response to the increased FFA levels in early HF, so as to decrease the SFA levels and possibly protect the HF.

### 2.5 Alteration of Gut Microbiota and Fecal Fatty Acids in Isoproterenol‐Induced HF Mice Model

**Figure 5:**
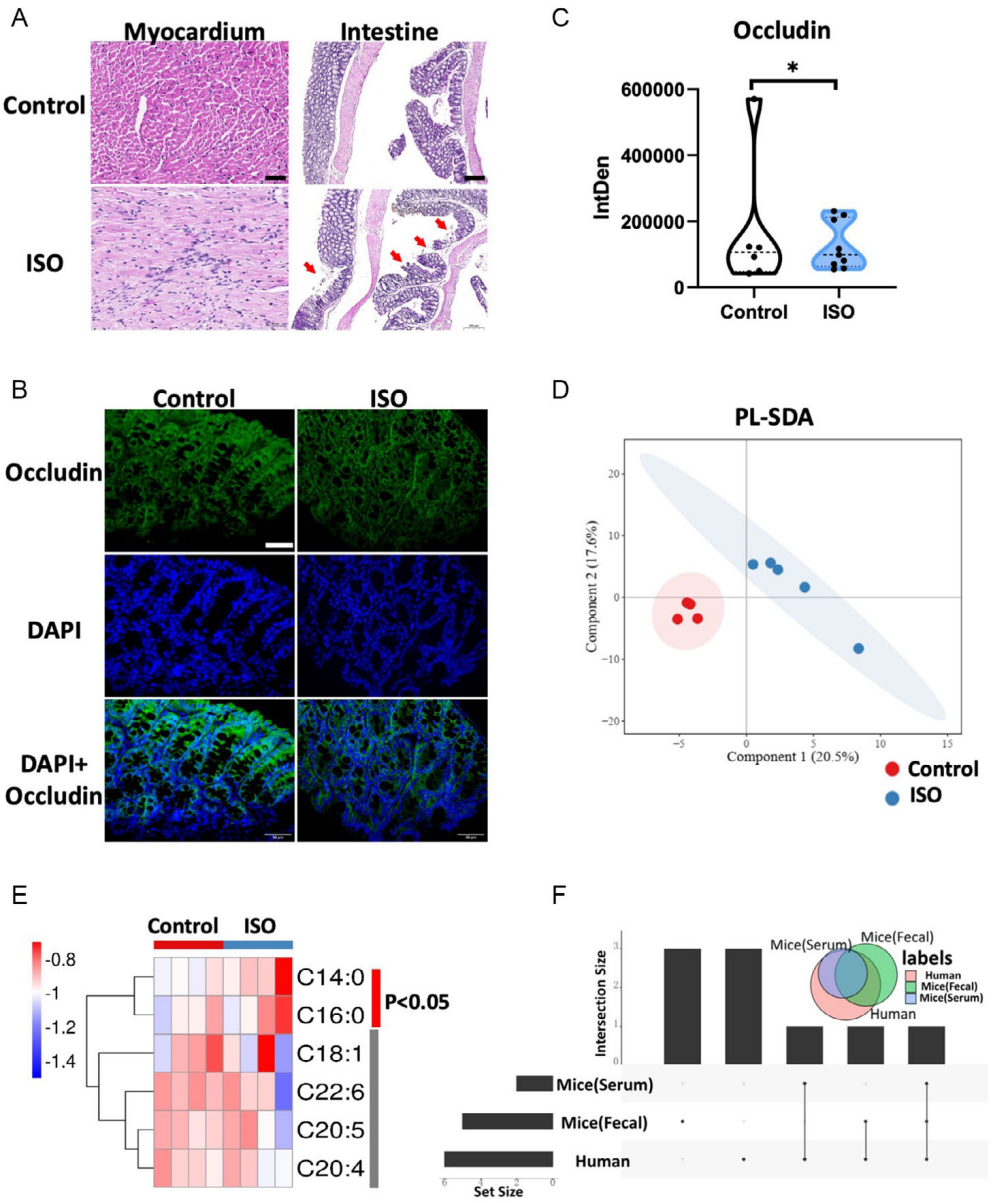
Alteration of fatty acids in isoproterenol‐induced HF mice model. A) H&E staining of the myocardium (scale bar: 50 μm) and intestine (scale bar: 200 μm) in control and ISO‐treated mice. B) Representative immunofluorescence images of occludin (green) in the intestinal epithelium of MI mice. Nuclei are stained with DAPI (blue), and scale bars are 50 μm. C) Quantification of the occludin immunostaining. D) Projection plots of serum FFAs from PLS‐DA for the control (blue dots) and ISO (red dots) group. E) Heatmap showing serum FFA levels in control and ISO group. F) UpSet Venn diagram showing the intersection of the significant differential FFAs in human serum and mouse serum and feces. *N* = 5 mice per group.

To further prove the gut microbial translocation and the *Clostridium*–FFA alteration in HF, isoproterenol (ISO)‐induced HF mice model was used. ISO (30 mg kg^−1^) was administered to the C57BL/6J mice subcutaneously for 2 weeks. Local inflammation of myocardium was observed in ISO group (**Figure** **5**A). Occludin expression (tight junction protein as the intestinal permeability marker) in the mucous membrane of intestinal epithelium was downregulated upon ISO induction (Figure 5B,C), further validating the altered intestinal permeability in HF.^[^ ^27^ ^]^ Fecal and serum SFAs were then assessed to show substantial differences of FFAs in ISO mice compared to control (Figure 5D; Figure S4, Supporting Information). FFA 14:0 and FFA 16:0 were statistically significantly increased in ISO‐induced mice model (Figure 5E), in accordance with human FFAs alteration pattern (Figure 5F).

**Figure 6:**
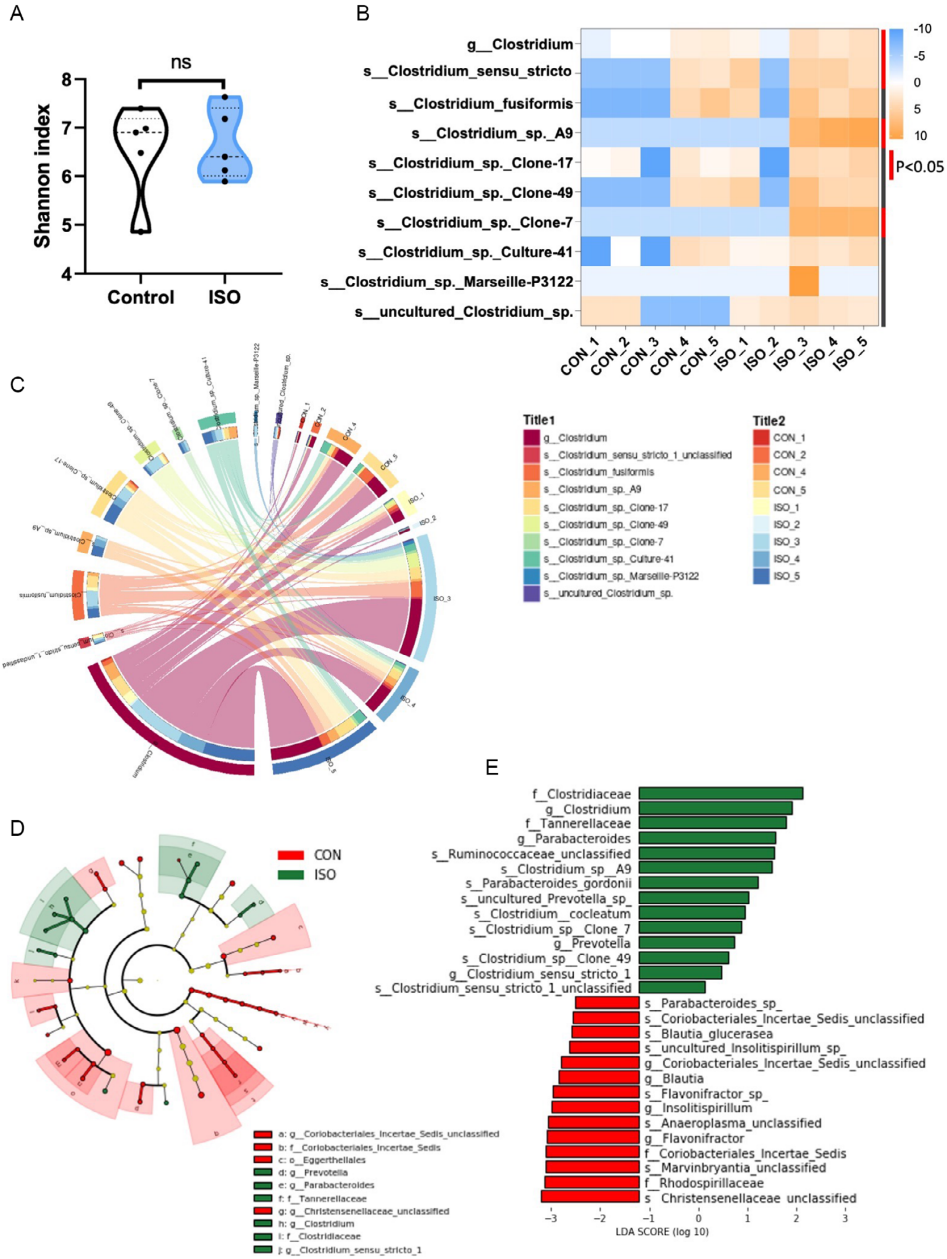
Alteration of gut microbiota in isoproterenol‐induced HF mice model. A) *α* diversity by 16s RNA in the control and ISO mice. B) Heatmap showing the *Clostridium* abundance at genus and species levels. C) Circos plot showing the composition of *Clostridium* between control and ISO. D) Cladogram generated from LDA and the LDA score. E) Showing the most differentially significant abundant taxa. *N* = 5 mice per group.

Fecal samples were then examined for bacterial community diversity via 16S rRNA gene amplicon sequencing. Although *α* diversity exhibited no obvious difference (**Figure** **6**A), the *Clostridium* abundance was significantly enriched both at genus and species levels in ISO‐induced mice (Figure 6B,C), suggesting that *Clostridium* reacted acutely upon HF onset. Linear discriminant analysis (LDA) indicated that genera belonging to the family *Clostridiaceae*, *Cannerellaceae*, and the genus *Clostridium* were significantly increased in ISO‐treated mice (Figure 6D,E).

### 2.6 Transplant of *C.sp* Rescued Decreased Cardiac Function While Increasing Circulating FFA 14:0 in ISO‐Induced HF Model

**Figure 7:**
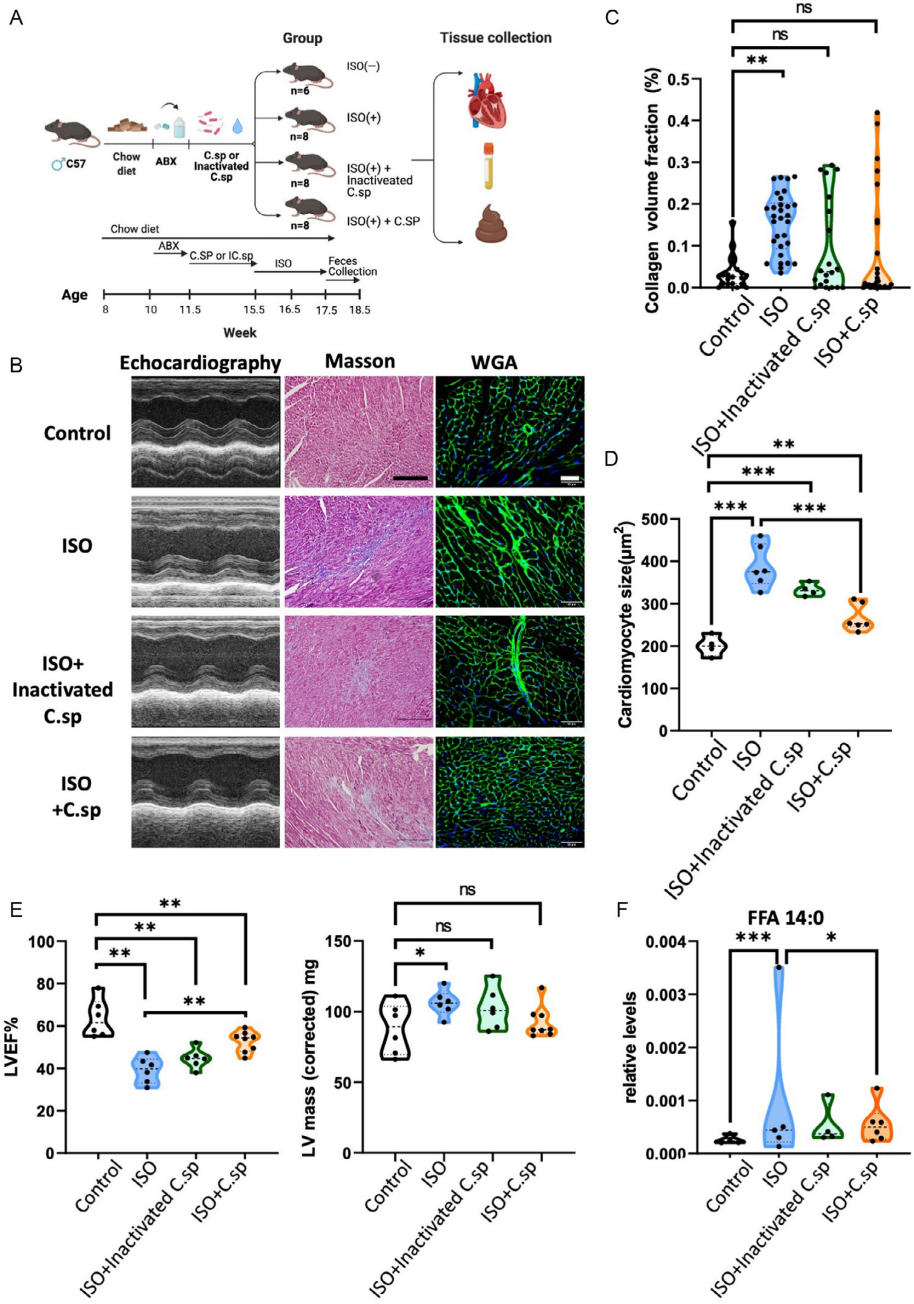
Transplant of *C.sp* rescued decreased cardiac function while increasing circulating FFA 14:0 in isoproterenol‐induced HF model. A) Flowchart of the animal study. *N* = 6–8 mice per group. B) Analysis of the myocardium from different groups using Masson staining (scale bar: 100 μm), WGA immunostaining (scale bar: 50 μm), and echocardiography. C) Quantification of the collagen volume fraction by Masson staining, in respective groups. D) Quantification of the cardiomyocyte size by WGA immunostaining in respective groups. E) Quantification of LVEF and corrected LV mass by echocardiography. WGA immunostaining in respective groups. F) Circulating FFA 14:0 levels in respective groups (ns, not significant; *, *P* < 0.05; **, *P* < 0.01; ***, *P* < 0.001).

To further clarify whether *C.sp* participated in the improvements of myocardial function in HF, we gave *C.sp* to C57BL/6J mice by gavage for 4 weeks after 10 d antibiotics cocktail treatment, and then treated with ISO for 2 weeks (**Figure** **7**A). Increased myocardial fibrosis in the myocardium of ISO‐treated mice was significantly alleviated by supplementing *C.sp* (Figure 7B,C), so is the cardiomyocyte cross‐sectional area evaluated by wheat germ agglutinin (WGA) staining (Figure 7B,D). However, the intestinal permeability was not mitigated in the gross morphology and occluding staining of the intestine (Figure S5A,B, Supporting Information). To assess the effect of *C.sp* on cardiac function, echocardiogram analysis was performed on control and ISO‐treated mice supplemented with *C.sp* or inactivated *C.sp*. Activated *C.sp* partially rescued myocardial function deterioration in ISO‐treated mice (Figure 7E). Moreover, the LV mass was not significantly increased as compared to the control in both *C.sp* or inactivated *C.sp* group (Figure 7E). At last, *C.sp* administration markedly abrogated FFA 14:0 levels in ISO‐treated mice (Figure 7F).

**Figure 8:**
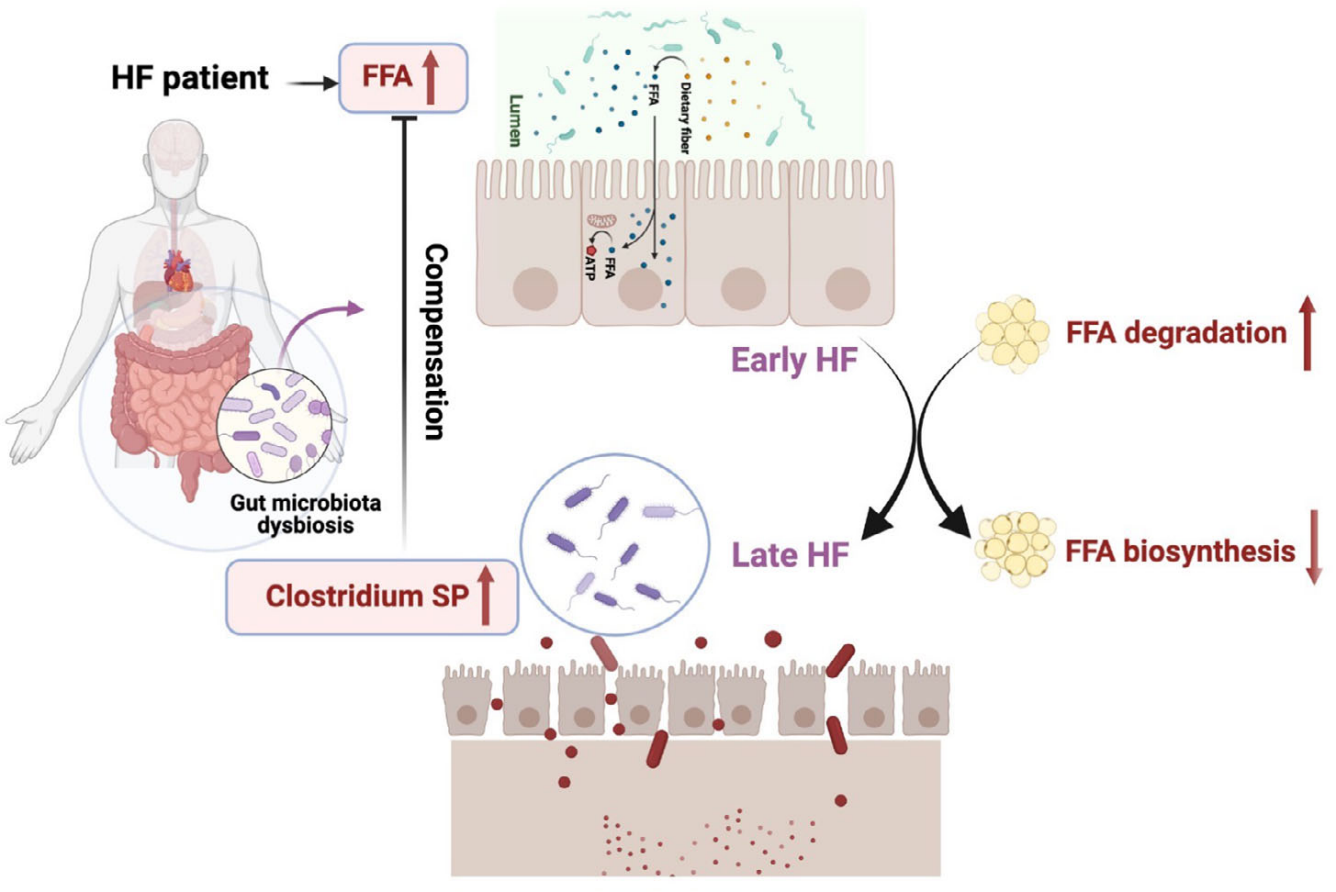
Proposed mechanism in the present study. Proposed mechanism in the present study suggesting that *Clostridium* abundance was enriched in the early and late phase of HF and further improved cardiac function by reducing the circulating SFAs levels.

In sum, in the present study, we identified gut microbiota alteration in both early and late HF patients, gene functions of which were enriched in FFA metabolism. *Clostridium* abundance was increased in the early phase of HF and further improved cardiac function by reducing the SFAs levels (**Figure** **8**).

## 3 Discussion

In this study, we have investigated the gut microbiota and its correlation to circulating FFA profile in early and late HF patients, as well as in ISO‐induced HF mice. By applying metagenomics analysis, we have identified enriched abundance of *Clostridium* in early and late HF patients, negatively correlating to SFA levels. KEGG analysis further indicated microbiota gene enrichment in FFA degradation in early HF, and decreased gene expression in FFA synthesis in late HF. At last, by supplementing *C.sp* to ISO‐induced HF mice, the cardiac function, fibrosis, and myocardial size were partially rescued, together with the reduced circulating FFA 14:0 levels. To the best of our knowledge, this is the first study to investigate the probiotic function of *C.sp* in HF by downregulation of circulating FFA levels.

Previous studies postulate that bowel wall edema and impaired intestinal barrier function in HF can promote bacterial translocation and inflammation, resulting in changes of composition and diversity of gut microbiota.^[^ ^27^ ^]^ Several small cohort studies have also shown that the alterations in gut microbial communities are present in HF patients. However, most of these studies are based on 16S rRNA gene sequencing, which could only identify proportions of bacterial taxa within the gut and lacks detailed individual gut microbiota species information. In the present study, we applied metagenomics and deep sequencing for better classification of the microbial community members and in‐depth detection of altered microbiota in species levels that previously could not be cultured in vitro. It is noteworthy that gut microbiota diversity and species alteration were more dysregulated in late than early HF. And the gene enrichment data by KEGG further indicate differential pathway regulation in FFA degradation and synthesis, suggesting a potential intercorrelation between gut microbes and circulating fatty acids. It is difficult to establish a causal relationship between gut microbiota and HF for the lack of fecal microbiota transplantation experiments. However, transplantation of *C.sp* in mouse models rescued ISO‐induced decreased cardiac function, which improves the involvement of *C.sp* in the progression of HF, at least partially.

The majority of SCFAs are generated through the breakdown of various polysaccharides in the gastrointestinal tract, absorbed in the colon, the mechanisms of which are well documented.^[^ ^20^ ^]^ Acetate is typically synthesized from pyruvate through the acetyl‐CoA by most gut microbiota, as well as via the Wood–Ljungdahl pathway. Propionate is derived from the succinate, acrylate, and propanediol pathways, while butyrate is predominantly produced through the phosphortransbutyrylase/butyrate kinase pathway and the butyryl‐CoA: acetate CoA transferase pathway.^[^ 32, 33 ^]^ Coculturing *Clostridium* sp. BS‐1 with *Clostridium* sp. BS‐7 has been shown to enhance SCFA production in in vitro studies, whereas *Clostridium* sp. S1 primarily mainly produces butyrate along with a minor amount of acetate.^[^ 34, 35 ^]^ The principal fermentation activity of *Clostridium* sp. LQ25 remains focused on the butyrate production pathway, evidenced by the expression of genes encoding acetyl‐CoA‐catalyzed enzymes.^[^ ^36^ ^]^ In addition, the fermentation of amino acids by gut microbiota contributes to the formation of branched‐chain fatty acids. *Clostridium* species are known to generate SCFAs, short‐branched fatty acids, and aromatic fatty acids, including indole propionate, various aryl propionate, isobutyrate, 2‐methylbutyrate, isovalerate, isocaproate, propionate, and butyrate.^[^ 37, 38 ^]^ *Clostridium* species significantly contribute to the host's pool of branched SCFAs by encoding metabolic enzymes such as porA, croA, hadB, and so on.^[^ ^37^ ^]^ Furthermore, *Clostridium* species, serving as a microbial hallmark of youth, are crucial in the biosynthesis of SCFAs in vivo.^[^ ^39^ ^]^ Fecal SCFAs levels, including acetate, propionate, butyrate, isobutyrate, and 2‐methylbutyrate, have been observed to increase markedly following the transplantation of *Clostridium symbiosum* in mice.^[^ ^39^ ^]^ Similarly, the levels of acetic, propionic, and butyric acids in mice treated with *Clostridium butyricum* are notably higher compared to control mice.^[^ ^40^ ^]^

The major novelty for the present study is that we have identified *C.sp* as a link between SFAs and myocardial function amelioration during HF progression. The probiotic intervention of *C.sp* via targeting downregulation of FFAs will further guide the treatment of HF in the field of gut microbiota‐based therapy. *C.sp* has previously been reported to exert beneficial effects in insulin resistance and antitumor therapy.^[^ 41, 42 ^]^ Our study has first identified the involvement of *C.sp* in myocardial function. It is hypothesized that the *C.sp* in the gut enriched in response to the increased FFA levels in HF, which, in turn, could orchestrate FFA metabolism and finally mitigate cardiac function deterioration. During the disease progression of HF, the increase of *C.sp* in the gut compensates the myocardial damage by regulating circulating FFAs; however, the host response of *C.sp* rise is insufficient to completely prevent HF progression.

The energy metabolism in HF patients, especially the glucose and fatty acids regulation has always been controversial. The glucose–fatty acid cycle theory postulates that there is competition between glucose and fatty acids for their oxidation.^[^ ^43^ ^]^ It has also been identified that cardiac pathological structural remodeling during HF also results in reprogramming of cardiac metabolic pathways, leading to an overall increased reliance on glucose metabolism and decrease in fatty acid oxidation.^[^ 44, 45 ^]^ As a result, the dysregulation of FFA has become a significant metabolic signature and is closely mechanistically related to cardiac contractility in the progression of HF.^[^ ^46^ ^]^ Yet, meanwhile, complex clinical data and limited knowledge about how FFAs affect cardiac function in HF patients mandate additional metabolic profiling and mechanism studies. In our analysis, although serum FFA levels were increased in late HF patients which is in accordance with previous speculation, the FFA levels in early HF patients remain relatively unaltered as compared to control. This is in accordance with the gene analysis of gut microbiota, which is enriched in FFA degradation in early HF, and decreased in FFA synthesis in late HF, suggesting host microbes alteration play a compensatory role during the pathological state of the body.

The present study evaluates circulating long‐chain FFA levels and their correlation to gut microbiota in HF patients. Circulating long‐chain fatty acids are absorbed into the fatty walls of the intestine villi and reassemble again into triglycerides instead of being directly released into the intestinal capillaries. Previous microbiota studies have been focusing on SCFAs, yet SCFA receptors were not readily detectable in the cardiac transcriptome. Therefore, additional mechanisms by which FFAs modulate cardiac function remain to be explored. In accordance with our results, recent study has also identified interaction of gut microbiota and circulating FFA.^[^ ^47^ ^]^ But the exact mechanisms are still not fully understood. Therefore, our study has provided further mechanistic insight that *C.sp* increase in response to the increased FFA levels in early HF, so as to decrease the SFA levels and possibly protect HF.

## 4 Our Study Has Several Limitations

Although we have studied three groups along the HF progression, the cohort size for each group is relatively limited, and the selection and observation bias could not be easily excluded. Besides, present investigation is agnostic to tissue source of circulating FFAs. Thus, further molecular and biochemical confirmation are also required to explain the exact metabolic pathway alterations. Large‐scale studies are also necessary to validate the interaction between gut microbiota and FFAs for disease phenotypes.

## 5 Conclusion

In summary, our results suggest that an altered gut microbiota in HF is associated with increased circulating FFA levels. Increased *C.sp* negatively correlates to circulating SFA levels both in HF patients and mice, which, in turn, orchestrates cardiac function. The present results suggest a *C.sp*–SFA–cardiac function axis in HF patients, which at least partially contributes to HF progression. Probiotic intervention with *C.sp* may bring a new breakthrough in the treatment of HF.

## 6 Experimental Section

6.1

6.1.1

##### Participants Enrollment

All participants were enrolled from the First Affiliated Hospital of Xi'an Jiaotong University, China between 2021 March and 2022 September. Three groups of subjects were recruited for these studies: No‐HF controls were defined by LVEF ≥50%, normal diastolic function, and no history of HF. Early‐stage HF was defined by LVEF < 50%, NT‐proBNP>200 ng mL^−1^, and NYHA grade II (slight limitation by shortness of breath or fatigue during moderate exertion or stress). And late HF was defined by LVEF < 40%, NT‐proBNP>200 ng mL^−1^, and NYHA grade III (having symptoms with minimal exertion that interfere with normal daily activity) (Figure 1A). The subjects were excluded if they: have familial lipid metabolism disorder; had chronic endocrine diseases; were undergoing hemodialysis for renal failure; had acute or chronic hepatitis with increased transaminase activities; suffered from cachexia; had malignant tumor; or refuse to participant in the study. The patients of HF with preserved ejection fraction or end‐stage HF with multiple organ dysfunction were also excluded. Demographic and biochemical information was obtained as previously described.^[^ 48, 49, 50, 51 ^]^ Echocardiography was performed by experienced clinicians. LVEF was measured by echocardiography using Simpson's method. Serum samples were collected from all patients recruited, of which fecal sample were collected from 32 control, 22 early HF, and 19 late HF. Written informed consent was obtained according to the Declaration of Helsinki, and was approved by the ethics committee, Xi'an Jiaotong University.

##### Serum FFA Profile Determination

For serum FFA profile studies, serum samples were collected from patients at diagnosis using the same protocol as previously described.^[^ 48, 49, 50, 51 ^]^ Venous blood was withdrawn after overnight fast and immediately centrifuged at 3000 rpm for 10 min at 4 °C. Serum samples were separated and stored at −80 °C and aliquots were thawed for further processing. The sample preparation, instrumentation, metabolic annotation, and data analysis procedure are referred in our previously published methods with minor modification.^[^ 48, 49, 50, 51, 52, 53, 54 ^]^ Test mixtures comprise a group of commercially available standards with a mass range across the system mass range used for the study samples. Internal standards were added to the test samples in order to monitor analytical variations during the entire sample preparation and analysis processes. To diminish analytical bias within the entire analytical process, the samples were analyzed in group pairs but the groups were analyzed randomly. The QC samples, calibrators, and blank samples were analyzed across the entire sample set. Mass spectrometry‐based quantitative metabolomics refers to the determination of the concentration of a substance in an unknown sample by comparing the unknown to a set of standard samples of known concentration.

##### Metagenomics and 16s RNA Sequencing

Stool samples from study participants and mice were collected and stored at −80 °C until further processing. Sample collection, DNA extraction, metagenomics and 16S RNA sequencing, and postsequencing processing were performed as previously described.^[^ 55, 56 ^]^ Sequencing libraries were generated using NEBNext UltraTM DNA Library Prep Kit for Illumina (NEB, USA) following manufacturer's recommendations and index codes were added to attribute sequences to each sample. Libraries were analyzed for size distribution by Agilent2100 Bioanalyzer and quantified using real‐time PCR. The clustering of the index‐coded samples was performed on a cBot Cluster Generation System according to the manufacturer's instructions. After cluster generation, the library preparations were sequenced on an Illumina HiSeq platform and paired‐end reads were generated. For data processing, adaptor contamination and low‐quality reads were discarded from the raw sequencing reads, and the remaining reads were filtered to eliminate human host DNA based on the human genome reference. All high‐quality reads from each sample were assembled to contigs by using metaSPAdes from SPAdes v3.10.0 package with default settings, and a nonredundant gene catalog was constructed by MetaGeneMark and CD‐HIT. Gene profile was calculated according to the high‐speed quantification tool Kallisto and normalized by gene length. Genes were annotated by blasting against the NCBI database using Diamond. The taxonomic composition was acquired by MEGAN and KO composition obtained using the KEGG Orthology‐Based Annotation System tool. The sum of relative abundance of all genes involved in a KEGG pathway was defined to be pathway abundance. Gene counts were calculated by counting the number of genes in each sample. Alpha diversity was performed on the basis of the gene profile of each sample according to the Shannon index. For 16s RNA sequencing, the abundance and diversity of intestinal flora in mice were determined using Illumina HiSeq sequencing (Novogene, Beijing, China), after amplification and purification of the V3–V4 region of bacterial 16s rRNA genes. The QIIME software package was used to conduct the bioinformatic analyses of the sequences. QIME was used to carry out alpha, beta diversity, and Principal Coordinates Analysis depending on the unweighted UniFrac distances.

##### Animal Model

All animals were given chow diet. After 2 week adaptation for the environmental changes, for the gut microbiota experiment, we start to treat mice with an antibiotics cocktail (ampicillin 1 g L^−1^ in drinking water, metronidazole 100 mg kg^−1^, neomycin 100 mg kg^−1^, and vancomycin 50 mg kg^−1^ by gavage) or 0.9% NaCl for 10 days. Then to transplant *C.sp*, all mice were fed with *C.sp*. (10^8^ CFU/mouse) or inactivated *C.sp* by gavage twice a week for 4 weeks. After 4 weeks of microbiota transplantation, 15.5 week old C57BL/6J mice were injected subcutaneously with ISO (30 mg kg^−1^ d^−1^, Sigma–Aldrich, MO, USA) or 0.9% NaCl for 14 d to induce HF model. Fresh fecal samples were collected from the mice and stored at −80 °C until use. After 1 week, all mice were euthanized by exsanguination under isoflurane. The serum samples and some tissues were harvested and fixed in paraffin or stored at −80 °C. Experimental protocols in our study were approved by the Institutional Ethics Committee for Animal Experiments of Xi'an Jiaotong University. All procedures conformed to the U.S. National Institutes of Health Guide for the Care and Use of Laboratory Animals. The manipulation of animals was performed in the Animal Center of Xi'an Jiaotong University. All mice were male and maintained in specific pathogen‐free environment. Mice aged 8 weeks were used and were maintained under a 12 h light/12 h dark cycle at 23 ± 1 °C and humidity (45–55%) with free access to food and water. Adult male C57BL/6J mice were purchased from Beijing Vital River Laboratory Animal Technology Co., Ltd.

##### Transthoracic Echocardiography

Transthoracic 2D M‐mode echocardiography was performed on days 7 and 14 postoperatively using Vevo 1100 (VisualSonics, Toronto, Canada) by an experienced technician blind to treatment allocation. LVEF was calculated as described previously.^[^ ^57^ ^]^

##### Histology and Immunofluorescence

Hematoxylin–eosin (H&E) and Masson's trichrome staining were performed by standard methods as described previously.^[^ ^58^ ^]^ Heart tissues fixed with 4% paraformaldehyde were embedded in paraffin and sectioned. Tissue sections were stained with H&E to determine the severity of myocardial inflammation. Collagen volume fraction of heart was assessed through Masson staining. Finally, sections were mounted using a xylene‐based mounting medium, and images were acquired using a light microscope (OLYMPUS, BX53). The cross‐sectional area of cardiomyocytes was measured in images captured in sections stained with 5 μM WGA (Sigma–Aldrich, MO, USA). Fixed colon tissues were embedded in paraffin, stained with occludin incubated with fluorescein isothiocyanate‐coupled secondary antibodies, and counterstained with DAPI. The stainings were examined under the fluorescence microscope (OLYMPUS, BX53).

##### Quantification of Size by Planimetry

The analysis of scar formation was performed using the noncommercial image processing software ImageJ (http://imagej.nih.gov/ij/). For this, the measure function of ImageJ was used on images of both Masson staining and WGA staining. The collagen volume fraction of heart was expressed as percent of the whole heart. Each group calculated more than 100 cells size randomly and was expressed by average.

##### Statistical Analysis

Data were presented as frequencies or percentages for categorical variables and mean ± SD for continuous variables, unless otherwise indicated. Simple *t*‐test was used to compare continuous variables which were in the normal distribution. Mann–Whitney *U* test was used to compare continuous variables which did not conform to the normal distribution. One‐way analysis of variance (ANOVA) was used to compare continuous variables of three or more independent (unrelated) groups. Two‐way ANOVA followed by Holm–Sidak test was used to evaluate the statistical significance of differences among three or more groups. The correlation analysis of FFA, gut microbiota, and clinical features was performed using Spearman's rank‐order correlation (R 3.5.1). KEGG enrichment analysis was performed using R 3.5.1 and iPath 3 (https://pathways.embl.de/). *P*‐value <0.05 was considered as significant.

## Conflict of Interest

The authors declare no conflict of interest.

## Author Contributions

Y.W., Z.Y., and J.S.: Designed experiments; Y.W., J.S., G.T., and M.G.: drafted the manuscript; J.S., M.G., J.L., W.X., B.L., C.W., X.Y., H.L., Y.X., L.L., and B.Z.: collected the clinical samples; J.S., Y.W., P.L., L.S., and M.G.: did the gut microbiota; F.F.A.: data analysis; G.T., Y.W., P.L., and X.Q.: did the animal experiments; G.T., Y.M., H.L., and J.S.: did the revision work. All authors revised, edited, and approved the final version of the manuscript.

## Supporting information

Supplementary Material

## Data Availability

The data that support the findings of this study are available from the corresponding author upon reasonable request.

## References

[1] C. W. Yancy , M. Jessup , B. Bozkurt , J. Butler , D. E. Casey , M. H. Drazner , G. C. Fonarow , S. A. Geraci , T. Horwich , J. L. Januzzi , M. R. Johnson , E. K. Kasper , W. C. Levy , F. A. Masoudi , P. E. McBride , J. J. McMurray , J. E. Mitchell , P. N. Peterson , B. Riegel , F. Sam , L. W. Stevenson , W. H. Tang , E. J. Tsai , B. L. Wilkoff , Circulation 2013, 128, 1810. 23741057 10.1161/CIR.0b013e31829e8807

[2] C. Y. Chen , M. A. Caporizzo , K. Bedi , A. Vite , A. I. Bogush , P. Robison , J. G. Heffler , A. K. Salomon , N. A. Kelly , A. Babu , M. P. Morley , K. B. Margulies , B. L. Prosser , Nat. Med. 2018, 24, 1225. 29892068 10.1038/s41591-018-0046-2 PMC6195768

[3] B. Lou , H. Wu , H. Ott , K. Bennewitz , C. Wang , G. Poschet , H. Liu , Z. Yuan , J. Kroll , J. She , J. Transl. Med. 2023, 21, 199. 36927819 10.1186/s12967-023-04050-5 PMC10018852

[4] L. Da Dalt , A. G. Cabodevilla , I. J. Goldberg , G. D. Norata , Cardiovasc. Res. 2023, 119, 1905. 37392421 10.1093/cvr/cvad100 PMC10681665

[5] D. Rodolico , G. G. Schiattarella , H. Taegtmeyer , JACC Heart Failure 2023, 11, 637. 37086246 10.1016/j.jchf.2023.02.007

[6] G. D. Lopaschuk , Q. G. Karwi , R. Tian , A. R. Wende , E. D. Abel , Circ. Res. 2021, 128, 1487. 33983836 10.1161/CIRCRESAHA.121.318241 PMC8136750

[7] L. Hooper , N. Martin , O. F. Jimoh , C. Kirk , E. Foster , A. S. Abdelhamid , Cochrane Database Syst. Rev. 2020, 5, Cd011737. 32428300 10.1002/14651858.CD011737.pub2 PMC7388853

[8] N. G. Forouhi , R. M. Krauss , G. Taubes , W. Willett , BMJ 2018, 361, k2139. 29898882 10.1136/bmj.k2139 PMC6053258

[9] K. Lotfi , A. Salari‐Moghaddam , M. Yousefinia , B. Larijani , A. Esmaillzadeh , Ageing Res. Rev. 2021, 72, 101467. 34560281 10.1016/j.arr.2021.101467

[10] P. M. Clifton , J. B. Keogh , Nutr. Metab. Cardiovasc. Dis. 2017, 27, 1060. 29174025 10.1016/j.numecd.2017.10.010

[11] M. Perna , S. Hewlings , Nutrients 2022, 15, 30. 36615688 10.3390/nu15010030 PMC9823926

[12] M. Ljubkovic , M. Gressette , C. Bulat , M. Cavar , D. Bakovic , D. Fabijanic , I. Grkovic , C. Lemaire , J. Marinovic , Diabetes 2019, 68, 1924. 31391173 10.2337/db19-0423

[13] G. Voros , J. Ector , C. Garweg , W. Droogne , J. Van Cleemput , N. Peersman , P. Vermeersch , S. Janssens , Circ. Heart Failure 2018, 11, e004953. 30562098 10.1161/CIRCHEARTFAILURE.118.004953

[14] O. J. Muller , M. B. Heckmann , L. Ding , K. Rapti , A. Y. Rangrez , T. Gerken , N. Christiansen , U. E. E. Rennefahrt , H. Witt , S. Gonzalez Maldonado , P. Ternes , D. M. Schwab , T. Ruf , S. Hille , A. Remes , A. Jungmann , T. M. Weis , J. S. Kreusser , H. J. Grone , J. Backs , P. Schatz , H. A. Katus , N. Frey , Cardiovasc. Res. 2019, 115, 1296. 30418544 10.1093/cvr/cvy274

[15] R. N. Lemaitre , B. McKnight , N. Sotoodehnia , A. M. Fretts , W. T. Qureshi , X. Song , I. B. King , C. M. Sitlani , D. S. Siscovick , B. M. Psaty , D. Mozaffarian , J. Am. Heart Assoc. 2018, 7, e010019. 30608197 10.1161/JAHA.118.010019 PMC6404213

[16] K. C. Bedi , N. W. Snyder , J. Brandimarto , M. Aziz , C. Mesaros , A. J. Worth , L. L. Wang , A. Javaheri , I. A. Blair , K. B. Margulies , J. E. Rame , Circulation 2016, 133, 706. 26819374 10.1161/CIRCULATIONAHA.115.017545 PMC4779339

[17] T. R. Matsuura , P. Puchalska , P. A. Crawford , D. P. Kelly , Circ. Res. 2023, 132, 882. 36996176 10.1161/CIRCRESAHA.123.321872 PMC10289202

[18] A. N. Carley , S. K. Maurya , M. Fasano , Y. Wang , C. H. Selzman , S. G. Drakos , E. D. Lewandowski , Circulation 2021, 143, 1797. 33601938 10.1161/CIRCULATIONAHA.120.052671 PMC8096711

[19] K. Makki , E. C. Deehan , J. Walter , F. Bäckhed , Cell Host Microbe 2018, 23, 705. 29902436 10.1016/j.chom.2018.05.012

[20] D. J. Morrison , T. Preston , Gut Microbes 2016, 7, 189. 26963409 10.1080/19490976.2015.1134082 PMC4939913

[21] T. Takeuchi , K. Kameyama , E. Miyauchi , Y. Nakanishi , T. Kanaya , T. Fujii , T. Kato , T. Sasaki , N. Tachibana , H. Negishi , M. Matsui , H. Ohno , Cell Metab. 2023, 35, 361.e9. 36652945 10.1016/j.cmet.2022.12.013

[22] L. Zhao , Y. Huang , L. Lu , W. Yang , T. Huang , Z. Lin , C. Lin , H. Kwan , H. L. X. Wong , Y. Chen , S. Sun , X. Xie , X. Fang , H. Yang , J. Wang , L. Zhu , Z. Bian , Microbiome 2018, 6, 107. 29903041 10.1186/s40168-018-0492-6 PMC6003035

[23] J. Miyamoto , M. Igarashi , K. Watanabe , S. I. Karaki , H. Mukouyama , S. Kishino , X. Li , A. Ichimura , J. Irie , Y. Sugimoto , T. Mizutani , T. Sugawara , T. Miki , J. Ogawa , D. J. Drucker , M. Arita , H. Itoh , I. Kimura , Nat. Commun. 2019, 10, 4007. 31488836 10.1038/s41467-019-11978-0 PMC6728375

[24] M. Kummen , C. C. K. Mayerhofer , B. Vestad , K. Broch , A. Awoyemi , C. Storm‐Larsen , T. Ueland , A. Yndestad , J. R. Hov , M. Troseid , J. Am. Coll. Cardiol. 2018, 71, 1184. 29519360 10.1016/j.jacc.2017.12.057

[25] K. A. Romano , I. Nemet , P. Prasad Saha , A. Haghikia , X. S. Li , M. L. Mohan , B. Lovano , L. Castel , M. Witkowski , J. A. Buffa , Y. Sun , L. Li , C. M. Menge , I. Demuth , M. König , E. Steinhagen‐Thiessen , J. A. DiDonato , A. Deb , F. Bäckhed , W. H. W. Tang , S. V. Naga Prasad , U. Landmesser , D. R. Van Wagoner , S. L. Hazen , Circ. Heart Failure 2023, 16, e009972. 36524472 10.1161/CIRCHEARTFAILURE.122.009972 PMC9851997

[26] W. H. W. Tang , D. Y. Li , S. L. Hazen , Nat. Rev. Cardiol. 2019, 16, 137. 30410105 10.1038/s41569-018-0108-7 PMC6377322

[27] A. Sandek , J. Bauditz , A. Swidsinski , S. Buhner , J. Weber‐Eibel , S. von Haehling , W. Schroedl , T. Karhausen , W. Doehner , M. Rauchhaus , P. Poole‐Wilson , H. D. Volk , H. Lochs , S. D. Anker , J. Am Coll. Cardiol. 2007, 50, 1561. 17936155 10.1016/j.jacc.2007.07.016

[28] O. Chioncel , A. P. Ambrosy , Eur. J. Heart Failure 2019, 21, 887. 10.1002/ejhf.1409 30623560

[29] T. Yang , M. M. Santisteban , V. Rodriguez , E. Li , N. Ahmari , J. M. Carvajal , M. Zadeh , M. Gong , Y. Qi , J. Zubcevic , B. Sahay , C. J. Pepine , M. K. Raizada , M. Mohamadzadeh , Hypertension 2015, 65, 1331. 25870193 10.1161/HYPERTENSIONAHA.115.05315 PMC4433416

[30] J. Pluznick , Gut Microbes 2014, 5, 202. 24429443 10.4161/gmic.27492 PMC4063845

[31] Y. Koretsune , T. Etoh , Y. Katsuda , T. Suetsugu , K. Kumeda , I. Sakuma , K. Eshima , M. Shibuya , S. I. Ando , N. Yokota , S. Goto , K. S. Pieper , J. Allu , A. K. Kakkar , G.‐A. Investigators , Circ. J. 2018, 83, 67. 30518731 10.1253/circj.CJ-18-0655

[32] C. Dion , E. Chappuis , C. Ripoll , Nutr. Metab. 2016, 13, 28. 10.1186/s12986-016-0086-x PMC4828828 27073407

[33] S. Ostadmohammadi , S. A. Nojoumi , A. Fateh , S. D. Siadat , F. Sotoodehnejadnematalahi , Acta Microbiol. Immunol. Hung. 2022, 69, 89. 10.1556/030.2022.01678 35397157

[34] B. S. Jeon , B. C. Kim , Y. Um , B. I. Sang , Appl. Microbiol. Biotechnol. 2010, 88, 1161. 20721546 10.1007/s00253-010-2827-5

[35] K. M. Lee , O. Choi , K. Y. Kim , H. M. Woo , Y. Kim , S. O. Han , B. I. Sang , Y. Um , Biotechnol. Lett. 2015, 37, 1837. 26026964 10.1007/s10529-015-1869-2

[36] S. Zhang , X. Zhang , Y. Yuan , K. Li , H. Liu , Sci. Total Environ. 2023, 855, 158911. 36152847 10.1016/j.scitotenv.2022.158911

[37] C. J. Guo , B. M. Allen , K. J. Hiam , D. Dodd , W. Van Treuren , S. Higginbottom , K. Nagashima , C. R. Fischer , J. L. Sonnenburg , M. H. Spitzer , M. A. Fischbach , Science 2019, 366, eaav1282. 31831639 10.1126/science.aav1282 PMC7141153

[38] Y. Liu , H. Chen , W. Van Treuren , B. H. Hou , S. K. Higginbottom , D. Dodd , Nat. Microbiol. 2022, 7, 695. 35505245 10.1038/s41564-022-01109-9 PMC9089323

[39] J. Lee , J. d’Aigle , L. Atadja , V. Quaicoe , P. Honarpisheh , B. P. Ganesh , A. Hassan , J. Graf , J. Petrosino , N. Putluri , L. Zhu , D. J. Durgan , R. M. Bryan , L. D. McCullough , V. R. Venna , Circ. Res. 2020, 127, 453. 32354259 10.1161/CIRCRESAHA.119.316448 PMC7415518

[40] D. Chen , D. Jin , S. Huang , J. Wu , M. Xu , T. Liu , W. Dong , X. Liu , S. Wang , W. Zhong , Y. Liu , R. Jiang , M. Piao , B. Wang , H. Cao , Cancer Lett. 2020, 469, 456. 31734354 10.1016/j.canlet.2019.11.019

[41] R. Gardlik , J. H. Fruehauf , IDrugs 2010, 13, 701. 20878592

[42] B. I. M. Salles , D. Cioffi , S. R. G. Ferreira , Diabetol. Metab. Syndr. 2020, 12, 98. 33292434 10.1186/s13098-020-00603-6 PMC7656736

[43] P. J. Randle , P. B. Garland , C. N. Hales , E. A. Newsholme , Lancet 1963, 1, 785. 13990765 10.1016/s0140-6736(63)91500-9

[44] S. Ravassa , T. Trippel , D. Bach , D. Bachran , A. Gonzalez , B. Lopez , R. Wachter , G. Hasenfuss , C. Delles , A. F. Dominiczak , B. Pieske , J. Diez , F. Edelmann , Eur. J. Heart Failure 2018, 20, 1290. 10.1002/ejhf.1194 29709099

[45] A. A. Gibb , B. G. Hill , Circ. Res. 2018, 123, 107. 29929976 10.1161/CIRCRESAHA.118.312017 PMC6023588

[46] A. A. Challa , E. D. Lewandowski , JACC Basic Transl. Sci. 2022, 7, 730. 35958686 10.1016/j.jacbts.2021.12.010 PMC9357564

[47] J. Folz , Y. T. Oh , I. Blazenovic , J. Richey , O. Fiehn , J. H. Youn , Mol. Nutr. Food Res. 2019, 63, e1900752. 31675161 10.1002/mnfr.201900752

[48] J. She , J. Feng , Y. Deng , L. Sun , Y. Wu , M. Guo , X. Liang , J. Li , Y. Xia , Z. Yuan , Dis. Markers 2018, 2018, 8. 10.1155/2018/5236267 PMC6304898 30627225

[49] J. She , Y. Deng , Y. Wu , Y. Xia , H. Li , X. Liang , R. Shi , Z. Yuan , Cardiovasc. Diabetol. 2017, 16, 97. 28789650 10.1186/s12933-017-0578-7 PMC5549379

[50] J. She , M. Guo , H. Li , J. Liu , X. Liang , P. Liu , B. Zhou , S. Liu , Y. Deng , B. Lou , C. Sun , Z. Yuan , Y. Wu , Clin. Sci. 2018, 132, 2135. 10.1042/CS20180247 PMC6365628 30190284

[51] J. She , Z. Yuan , Y. Wu , J. Chen , J. Kroll , Mol. Metab. 2018, 8, 189. 29203238 10.1016/j.molmet.2017.11.006 PMC5985015

[52] Y. Qiu , G. Cai , M. Su , T. Chen , X. Zheng , Y. Xu , Y. Ni , A. Zhao , L. X. Xu , S. Cai , W. Jia , J. Proteome Res. 2009, 8, 4844. 19678709 10.1021/pr9004162

[53] J. H. Wang , W. L. Chen , J. M. Li , S. F. Wu , T. L. Chen , Y. M. Zhu , W. N. Zhang , Y. Li , Y. P. Qiu , A. H. Zhao , J. Q. Mi , J. Jin , Y. G. Wang , Q. L. Ma , H. Huang , D. P. Wu , Q. R. Wang , Y. Li , X. J. Yan , J. S. Yan , J. Y. Li , S. Wang , X. J. Huang , B. S. Wang , W. Jia , Y. Shen , Z. Chen , S. J. Chen , Proc. Natl. Acad. Sci. U. S. A. 2013, 110 17017. 24082129 10.1073/pnas.1315558110 PMC3801077

[54] Y. Ni , Y. Qiu , W. Jiang , K. Suttlemyre , M. Su , W. Zhang , W. Jia , X. Du , Anal. Chem. 2012, 84, 6619. 22747237 10.1021/ac300898h

[55] H. W. Xiao , M. Cui , Y. Li , J. L. Dong , S. Q. Zhang , C. C. Zhu , M. Jiang , T. Zhu , B. Wang , H. C. Wang , S. J. Fan , Microbiome 2020, 8, 69. 32434586 10.1186/s40168-020-00845-6 PMC7241002

[56] L. H. Quan , C. Zhang , M. Dong , J. Jiang , H. Xu , C. Yan , X. Liu , H. Zhou , H. Zhang , L. Chen , F. L. Zhong , Z. B. Luo , S. M. Lam , G. Shui , D. Li , W. Jin , Gut 2020, 69, 1239. 31744910 10.1136/gutjnl-2019-319114

[57] S. K. Verma , P. Krishnamurthy , D. Barefield , N. Singh , R. Gupta , E. Lambers , M. Thal , A. Mackie , E. Hoxha , V. Ramirez , G. Qin , S. Sadayappan , A. K. Ghosh , R. Kishore , Circulation 2012, 126, 418. 22705886 10.1161/CIRCULATIONAHA.112.112185 PMC3422741

[58] D. Otali , J. Fredenburgh , D. K. Oelschlager , W. E. Grizzle , Biotech. Histochem. 2016, 91, 309. 27149658 10.1080/10520295.2016.1179342 PMC5338041

