# Supplementary material for: Gut Microbiota Regulate Saturated Free Fatty Acid Metabolism in Heart Failure
Source: Small Sci. 2024 Jul 8;4(9):2300337. doi: 10.1002/smsc.202300337 (PMC11935106; doi:10.1002/smsc.202300337)
Supplement: Supplementary file 1 — Supplementary Material [file SMSC-4-2300337-s001.pdf]

Supplementary Figures

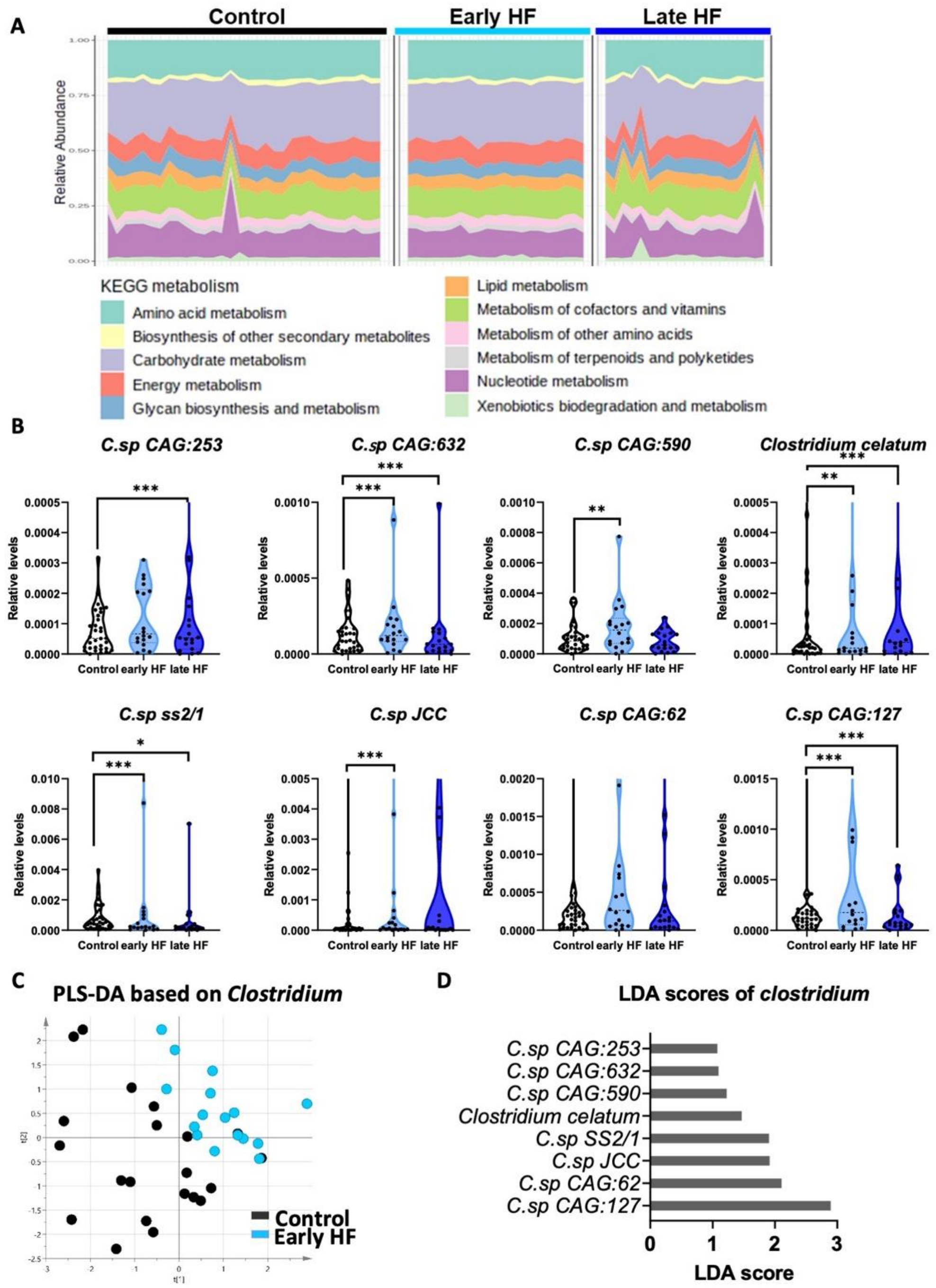

Supplementary Figure 1. Individual FFA levels were altered in HF patients. A. Stack plots of the KEGG metabolism in control, early and late HF patients. B. Significantly altered *Clostridium* at the species levels in control, early and late HF. (*, P<0.05; **, P<0.01; ***, P<0.001) C. Projection plots of *Clostridium* species from PLS-DA for the control (black dots) and early HF (light blue dots). D. LDA scores of the *Clostridium*.

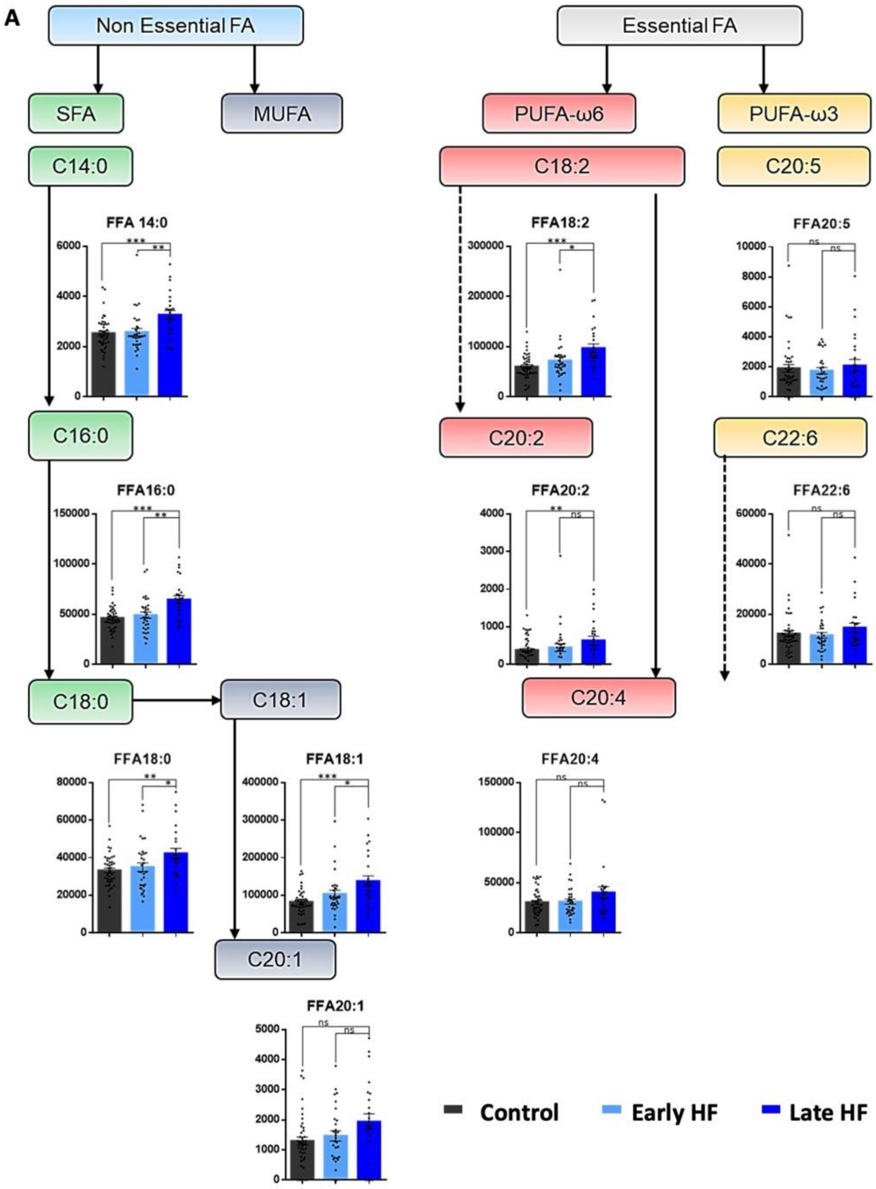

Supplementary Figure 2. Individual FFA levels were altered in HF patients. (ns, not significant. *, P<0.05. **, P<0.01. ***, P<0.001.).

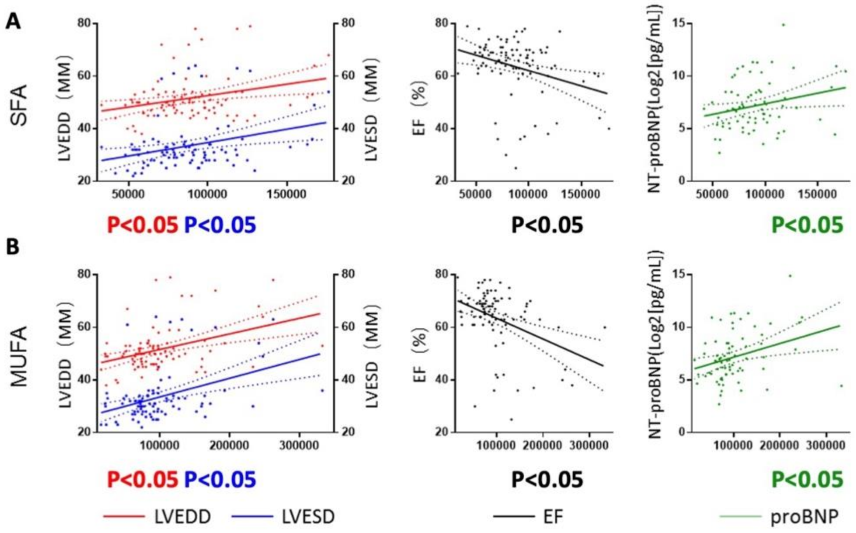

Supplementary

Figure 3. Correlation between SFA, MUFA, PUFA and cardiac function.

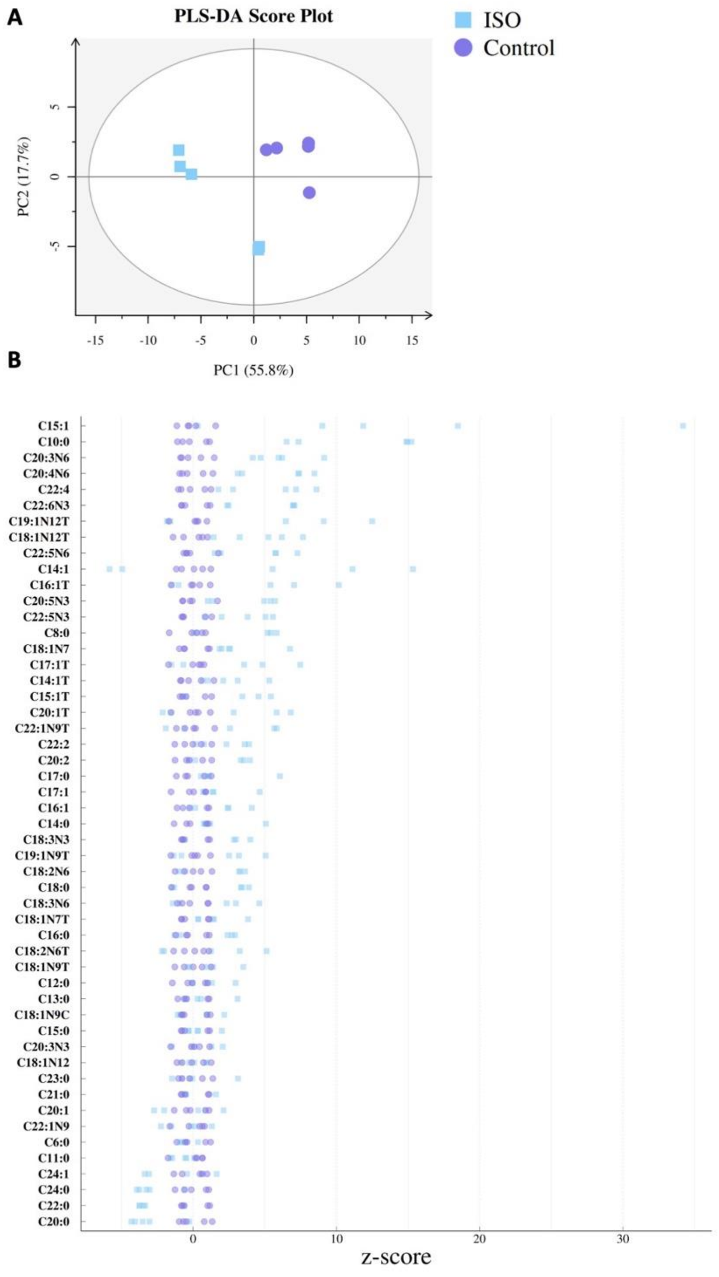

Supplementary Figure 4. PLS-DA and Z score of the Fecal FFA metabolites in ISO mice as compared to control.

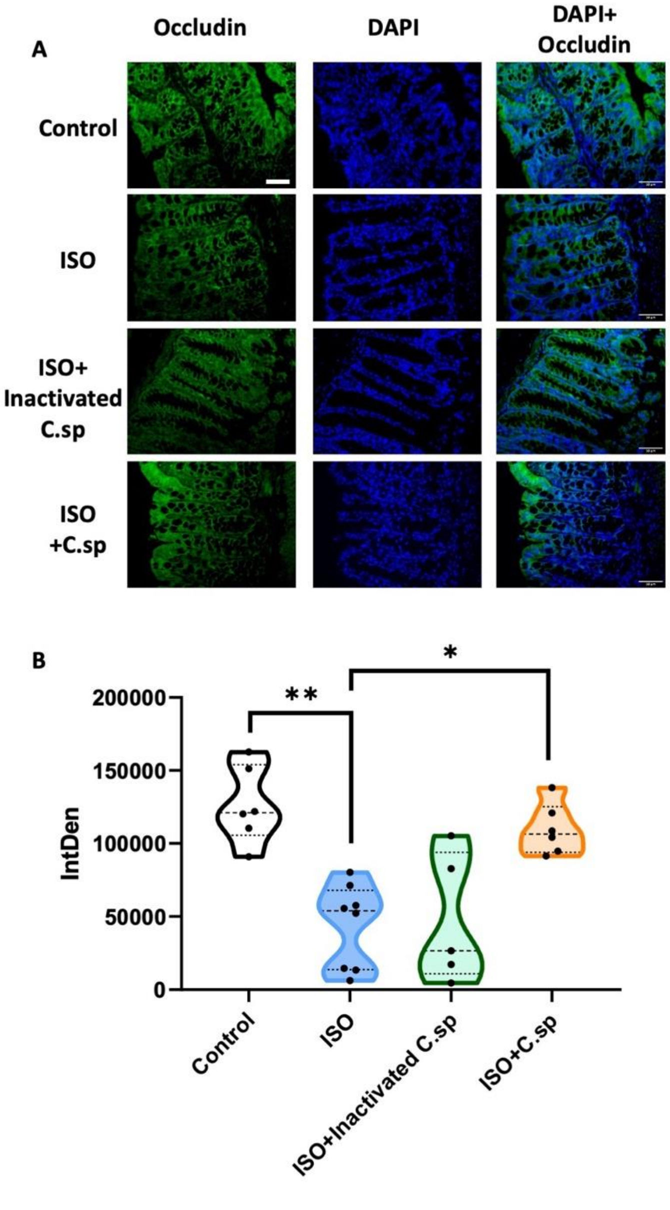

Supplementary Figure 5. Occludin staining of the intestine in HF and control mice with *Clostridium sp.* or inactivated *Clostridium sp.* transplant. A. Representative immunofluorescence images of occludin (green) in the intestinal epithelium of MI mice. Nuclei are stained with DAPI (blue), and scale bars are 50 μm. B. Quantification of the Occludin immunostaining. N=6-8 mice per group.

Supplementary Table 1. List of Free Fatty acids being tested

	Name	Abbreviation	KEGG ID
	Myristic acid	FFA 14:0	C06424
SFA	Palmitic acid	FFA 16:0	C00249
	Stearic acid	FFA 18:0	C01530
MUFA	Oleic acid	FFA 18:1	C00712
	Eicosenoic acid	FFA 20:1	C16526
	linoleic acid	FFA 18:2	C01595
PUFA-ω6	Octadecadienyl Acetate	FFA 20:2	C16525
	arachidonic acid	FFA 20:4	C00219
PUFA-ω3	Abietic acid	FFA 20:5	C06428
	Docosahexaenoic acid	FFA 22:6	C06429
